# SH003 reverses drug resistance by blocking signal transducer and activator of transcription 3 (STAT3) signaling in breast cancer cells

**DOI:** 10.1042/BSR20170125

**Published:** 2017-11-15

**Authors:** Hye-Sook Seo, Jin Mo Ku, Hee-Jae Lee, Jong-Kyu Woo, Chunhoo Cheon, Mia Kim, Bo-Hyoung Jang, Yong Cheol Shin, Seong-Gyu Ko

**Affiliations:** 1 Laboratory of Clinical Biology and Pharmacogenomics and Center for Clinical Research and Genomics, College of Korean Medicine, Kyung Hee University, 26 Kyungheedae-ro, Dongdaemun-gu, Seoul 02447, Republic of Korea; 2 Department of Science in Korean Medicine, Graduate School, Kyung Hee University, 26 Kyungheedae-ro, Dongdaemun-gu, Seoul 02447, Republic of Korea; 3 College of Veterinary Medicine, Seoul National University, 1, Gwanak-ro, Gwanak-gu, Seoul 08826, Republic of Korea; 4 Department of Cadiovascular and Neurologic disease (Stroke center), College of Korean Medicine, Kyung Hee University, 26 Kyungheedae-ro, Dongdaemun-gu, Seoul 02447, Republic of Korea

**Keywords:** breast cancer, drug resistance, multi-drug resistance 1, signal transducer and activator of transcription 3, SH003

## Abstract

Overcoming drug resistance is an important task for investigators and clinician to achieve successful chemotherapy in cancer patients. Drug resistance is caused by various factors, including the overexpression of P-glycoprotein (P-gp, MDR1). The development of new, useful compounds that overcome drug resistance is urgent. SH003 is extracted from the mixture of three different herbs, and its anticancer effect has been revealed in different cancer cell types. In the present study, we investigated whether SH003 is able to reverse drug resistance using paclitaxel-resistant breast cancer cells (MCF-7/PAC). In our experiments, SH003 significantly decreased cell growth and colony formation in MCF-7/PAC cells and parental MCF-7 cells. This growth inhibition was related to the accumulation of cells in the sub-G_0_/G_1_ apoptotic population and an increase in the number of apoptotic cells. SH003 reduced the mRNA expression of multidrug resistance 1 (MDR1) and multidrug resistance-associated proteins (MRPs) in MCF-7/PAC cells. SH003 also down-regulated the expression of P-gp. SH003 reversed drug efflux from MCF-7/PAC cells, resulting in rhodamine123 (Rho123) accumulation. Inhibition of drug resistance by SH003 is related to the suppression of the signal transducer and activator of transcription 3 (STAT3) signaling pathway. SH003 decreased STAT3 activation (p-STAT3) and its nuclear translocation and inhibited the secretion of VEGF and MMP-2, which are STAT3 target genes. An STAT3 inhibitor, JAK inhibitor I and an HIF-1α inhibitor decreased cell growth in MCF-7 and MCF-7/PAC cells. Taken together, these results demonstrate that SH003 can overcome drug resistance, and SH003 might be helpful for chemotherapy in cancer patients.

## Introduction

Overcoming drug resistance is an important task for investigators and clinician to achieve successful chemotherapy in cancer patients. Drug resistance concerns patients who develop pre-existing and resistance-mediating factors before receiving chemotherapy (intrinsic resistance) or who were initially sensitive to the chemotherapy but who developed resistance during treatment (acquired resistance) [1,2]. Numerous advanced techniques have increased our ability to identify novel genes and signaling pathways that are related to tumor responsiveness to a particular drug treatment [1]. Several mechanisms of overcoming drug resistance have been developed to cure cancer [3].

Drug resistance is developed due to drug inactivation, drug target alteration, drug efflux, DNA damage repair, cell death inhibition, and the epithelial–mesenchymal transition (EMT) [4]. Among these factors, drug efflux-mediated resistance due to the overexpression of ABC transporters is the one that most frequently occurs [5]. ABC transport molecules, which are expressed on the plasma membrane and on the membranes of cellular vesicles, have important physiologic functions and affect the transport of chemotherapeutic reagents in humans [5,6]. Currently, there are 48 known transporters in the ABC family, and 13 of the ABC transporters are related to tumor drug resistance, including P-glycoprotein (P-gp, MDR1/ABCB1), multidrug resistance proteins (MRPs/ABCCs), and breast cancer resistance protein (BCRP/ABCG2) [5,6]. P-gp (MDR1) normally protects several organs from toxic compounds, preventing them from entering the cytosol and discharging them to the cell exterior [6]. However, in patients with tumors, P-gp (MDR1) ejects chemotherapeutic agents out of the cells, decreasing their efficacy. P-gp (MDR1) is overexpressed in cancer cells and is one of the main barriers to successful chemotherapy treatments for cancer [6].

SH003 is extracted from the mixture of three different herbs [*Astragalus membranaceus* (Am), *Angelica gigas* (Ag), and *Trichosanthes kirilowii* Maximowicz (Tk)] [7]. Anticancer effects of herbal extracts from Am, Ag, and Tk have been revealed in different cancer cell types such as leukemia, hepatocellular carcinoma, colon cancer, non-small-cell lung cancer, and gastric cancer cells [7–14]. Furthermore, extracts from a mixture of Am and Ag have been shown to affect various diseases including hematologic disorders or endocrine disorders [15–17]. According to our previous report, SH003 showed anticancer effects on different breast cancer cells without affecting normal epithelial cell viability, both *in vitro* and *in vivo* [7]. Moreover, SH003 suppresses MDA-MB-231 cell growth and metastasis by inhibiting STAT3–IL-6 pathway [7]. These results suggest that SH003 may be useful chemotherapeutic agent to treat breast cancer.

STAT3 is a cytoplasmic transcription factor that mediates extracellular signaling to the nucleus controlling fundamental functions such as cell proliferation, apoptosis, differentiation, immune responses, and angiogenesis [18]. STAT3 is abnormally expressed in pathological situations such as cancer [19]. Upon ligand binding, STAT3 is activated, resulting in dimerization, translocation to the nucleus, binding to DNA response elements, and the induction of transcription of genes. Cancer cells expressing constitutively activated STAT3 are more resistant to apoptosis and chemotherapy [19].

In the present study, we investigated whether SH003 reverses drug resistance and the mechanism of action. For this purpose, we tested the effects of SH003 on proliferation and apoptosis of MCF-7 cells and paclitaxel-resistant MCF-7/PAC cells. We analyzed whether SH003 recovers cells from Paclitaxel resistance, resulting in down-regulation of P-gp (MDR1) expression. We also verified whether SH003 inhibits the STAT3 signaling pathway, leading to the suppression of breast cancer development and drug resistance. Because we report here that SH003 overcomes drug resistance, SH003 might be helpful for chemotherapy in cancer patients.

## Materials and methods

### Preparation of SH003

SH003 consists of Am, Ag, and Tk that is based on the principle of the traditional medicine. Herbal composition of SH003 is *Astragalus membranaceus* (Am), *Angelica gigas* (Ag), *Trichosanthes kirilowii* Maximowicz (Tk) = 1:1:1 (proportion). All extracts were provided from Hanpoong Pharm and Foods company (Jeonju, Republic of Korea) manufactured by the Good Manufacturing Product (GMP). Dried extracts were dissolved in 30% ethanol to prepare a stock solution of 20 mg/ml. The stock solution was stored at −80°C.

### Compounds

HIF-1α inhibitor (EF-24), 7-aminoactinomycin D (7-AAD), rhodamine 123, and nicardipine were purchased from Sigma Chemical Co. (St. Louis, MO, U.S.A.). These compounds were dissolved in dimethyl sulfoxide (DMSO) or ethanol, and the final concentration of DMSO or ethanol in the controls and in each sample did not exceed 0.1%. We found that 0.1% DMSO or ethanol did not affect the cell growth rate compared with 0% DMSO or ethanol (no treatment) in breast cancer cells (data not shown). The STAT3 inhibitor (S3I-201) was obtained from Calbiochem (San Diego, CA, U.S.A.). JAK inhibitor I was purchased from Santa Cruz Biotechnology, Inc. (Santa Cruz, CA, U.S.A.). Annexin V, Alexa Fluor® 488 Conjugate was obtained from Thermo Fisher Scientific Korea (Seoul, Korea). An EZ-western chemiluminescent detection kit was purchased from Daeillab Service Co. (Seoul, Korea).

### Cell cultures

MCF-7 (ATCC, American Type Culture Collection, Manassas, VA, U.S.A.) and MCF-7/PAC cells were cultured in Dulbecco’s modified Eagle’s medium (DMEM), containing 50 U/ml penicillin, 50 mg/ml streptomycin, and 10% fetal bovine serum (FBS; Welgene, Daegu, Korea) at 37°C in an atmosphere of 5% CO_2_. MCF-7/PAC cell line is established in our laboratory. To establish paclitaxel-resistant MCF-7/PAC cells, we treated MCF-7 cells with paclitaxel under rising concentrations (1–10 nM). When MCF-7 cells with paclitaxel are fully grown in Petri dishes under lower concentration, we cultured cells with higher concentration of paclitaxel. Surviving cells from paclitaxel treatment are established as MCF-7/PAC cell line.

### Antibodies

Primary antibodies directed against cleaved caspase-8, poly(ADP-ribose) polymerase (PARP), and P-gp (MDR1) were purchased from Cell Signaling Technology, Inc. (Danvers, MA, U.S.A.). Primary antibodies directed against STAT3 and phospho-STAT3 (Tyr705), cyclin D1 were obtained from Upstate-Millipore (Billerica, MA, U.S.A.). The anti-tubulin antibody was from Sigma Chemical Co. Horseradish peroxidase (HRP)-conjugated secondary antibodies (mouse and rabbit) were purchased from Calbiochem, and anti-goat secondary antibody was from Jackson ImmunoResearch (West Grove, PA, U.S.A.).

### MTT assay

MCF-7 and MCF-7/PAC cells were seeded in 96-well culture plates at a density of 3 × 10^3^ cells/well and incubated for 24 h at 37°C. Then, they were treated with paclitaxel (0–1 µM), SH003 (0–500 µg/ml), STAT3 inhibitor (0–500 µM), JAK inhibitor I (0–10 µM), or HIF-1α inhibitor (0–100 µM) for 48 or 72 h. After incubation, MTT reagents (0.5 mg/ml) were added to each well, and the plates were incubated in the dark at 37°C for 2 h. At the end of the incubation period, the medium was removed, the resulting formazan was dissolved in DMSO, and the optical density was measured at 570 nm using an ELISA plate reader.

### Clonogenic survival assay

For the colony formation assay, MCF-7 and MCF-7/PAC cells were seeded in six-well culture plates at a density of 5 × 10^2^ cells/well. After overnight incubation, the cells were treated with different concentrations of SH003 (0–1000 µg/ml) or vehicle and maintained for 10 days at 37°C. The cells were fed every 3 days by removing the old medium and adding fresh medium containing SH003. Finally, the plates were stained with hematoxylin, and colony numbers were counted.

### Cell cycle analyses by flow cytometry

MCF-7 and MCF-7/PAC cells treated with SH003 (0–500 µg/ml) were harvested with 0.25% trypsin and washed once with phosphate-buffered saline (PBS). After the cells were centrifuged, they were fixed in cold 95% ethanol with 0.5% Tween-20 and stored at −20°C for at least 30 min. The cells were incubated in 50 μg/ml of propidium iodide (PI) (including 1% sodium citrate and 50 μg/ml RNase A) at room temperature in the dark for 30 min. The analysis of apoptotic cells was performed with a FACScan flow cytometer (Becton Dickinson, Mountain View, CA, U.S.A.), and the data were analyzed using CellQuest software.

### Annexin V/7AAD apoptosis assay

Apoptotic cell death was measured by an annexin V-FITC and 7-AAD assay. Cells were stained with annexin V-FITC and 7-AAD for 30 min at room temperature in the dark. The apoptotic cell population was analyzed with a FACSCalibur^TM^ flow cytometer by measuring the signal in the FL-1 and FL-3 channels.

### Western blot analysis

Cells were lysed in modified RIPA buffer [150 mM NaCl, 1% NP-40, 0.5% deoxycholate, 0.1% SDS, 50 mM Tris (pH 8.0), 1 mM EDTA, 1 mM phenylmethylsulfonyl fluoride (PMSF), 1 mM NaF, 1 mM Na_3_VO_4_, and a protease inhibitor mixture]. The lysates were cleared by centrifugation at 13000 ***g*** for 15 min, and the supernatants were collected. The protein concentration was quantified using a Bio-Rad Bradford protein assay (Bio-Rad, Hercules, CA, U.S.A.). Equal amounts of protein lysates were used for Western blot analysis with the indicated antibodies. Immunoreactive protein bands were detected with an EZ-Western Detection kit.

### Immunocytochemistry

MCF-7 and MCF-7/PAC cells (2 × 10^4^ cells/well) were seeded in eight-well chamber slides, incubated for 24 h at 37°C, and treated with SH003 (500 µg/ml) for another 24 h. The cells were fixed with 4% paraformaldehyde for 30 min and treated with 3% hydrogen peroxide (H_2_O_2_) in methanol for 20 min to quench endogenous peroxidase activity. The cells were washed with PBS, blocked with 5% BSA in PBS for 1 h, and incubated with an anti-STAT3 primary antibody (1:100 dilution) overnight at 4°C. After the cells were washed with PBS, they were incubated with anti-rabbit biotin-conjugated secondary antibody for 1 h at room temperature. Then, the cells were treated with Vectastain ABC reagent (Vector Laboratories, Inc. Burlingame, CA, U.S.A.) for 30 min at 4°C and stained with diaminobenzidine tetrachloride (DAB) and hematoxylin. The cells were mounted with mounting medium and subsequently analyzed by microscopy.

### RNA extraction and reverse transcription-polymerase chain reaction (RT-PCR)

Whole cell lysates under diverse conditions were prepared by washing with ice-cold PBS. Total RNA was isolated using TRIzol reagent (iNtRON Biotechnology, Seong-Nam, Korea). Total RNA was treated with 2 units of RNase-free DNase at 37°C for 30 min, extracted with phenol/chloroform/isopropanol, and precipitated with ethanol. The RNA concentration was determined by measuring the absorbance at 260 nm using a NanoDrop 2000 spectrophotometer (ThermoFisher Scientific, Wilmington, DE, U.S.A.), and the ratio of absorbance at 260 and 280 nm was 1.8 or higher. cDNA was synthesized from 2 μg of total RNA as a template in 20 μl of reaction mixture containing 5× first strand buffer, 0.1 M DTT, 10 mM dNTPs, and 200 units of M-MLV reverse transcriptase (iNtRON Biotechnology). cDNA was incubated at 42°C for 1 h and inactivated at 95°C for 5 min. After inactivation, the cDNA was stored at −20°C until use. RT-PCR was performed by coamplification of the genes with a reference gene by use of the cDNA template and corresponding gene-specific primer sets. The primer sequences are shown in Table 1. PCR was conducted in a total volume of 25 μl containing 5 μl of cDNA solution and 25 μM sense primers, 25 μM antisense primers, 1× PCR buffer, 2.5 mM MgCl_2_, and 2.5 units of Taq DNA polymerase (TaKaRa Korea, Seoul, Korea). The sequencing involved 30 cycles at 94°C for 45 s for denaturation, 58°C for 45 s for annealing, and 72°C for 45 s for extension. The resulting PCR products were resolved on 1% agarose gels containing ethidium bromide.

**Table 1:** Primer sequences

Type	Primer name	Sequences	
Human	MDR1	Forward	5′-CCC ATC ATT GCA ATA GCA GG-3′
		Reverse	5′-GTT CAA ACT TCT GCT CCT GA-3′
Human	MRP1	Forward	5′-GCC GAA GGA GAG ATC ATC-3′
		Reverse	5′-AAC CCG AAA ACA AAA CAG G-3′
Human	MRP2	Forward	5′-CGA CCC TTT CAA CAA CTA CTC-3′
		Reverse	5′-CAC CAG CCT CTG TCA CTT C-3′
Human	MRP3	Forward	5′-GTG GGG ATC AGA CAG AGA T-3′
		Reverse	5′-TAT CGG CAT CAC TGT AAA CA-3′
Human	MRP4	Forward	5′-GCT CAG GTT GCC TAT GTG CT-3′
		Reverse	5′-CGG TTA CAT TTC CTC CTC CA-3′
Human	MRP5	Forward	5′-CAG CCA GTC CTC ACA TCA-3′
		Reverse	5′-GAA GCC CTC TTG TCT TTT TT-3′
Human	MRP6	Forward	5′-AGG AGG CCC GAG CTT AGA C-3′
		Reverse	5′-CCT GCC CTA TTG GAT GCT GT-3′
Human	MRP9	Forward	5′-ATG CGG TTG TCA CTG AAG-3′
		Reverse	5′-GTT GCC TCA TCC ATA ATA AGA AT-3′
Human	GAPDH	Forward	5′-CGT CTT CAC CAC CAT GGA GA-3′
		Reverse	5′-CGG CCA TCA CGC CAC AGT TT-3′

RT-PCR was performed by coamplification of the genes with a reference gene by use of the cDNA template and corresponding gene-specific primer sets

### Measurement of VEGF and MMP-2 secreted from MCF-7 and MCF-7/PAC cells by ELISA

To assess the level of VEGF and MMP-2 in the MCF-7 and MCF-7/PAC cell supernatants, the cells were treated with SH003 (0–500 µg/ml). After 24 h, the media were collected, centrifuged to remove the cellular debris, and stored at −70°C until assayed for VEGF and MMP-2. The amount of VEGF and MMP-2 secreted into the culture medium was measured by ELISA according to the manufacturer’s instructions (R&D Systems, Minneapolis, MN, U.S.A.). Briefly, 96-well plates were coated with capture antibody in ELISA coating buffer and incubated overnight at 4°C. The plates were then washed with PBS with 0.05% Tween 20 (PBS-T) and subsequently blocked with 10% FBS in PBS for 1 h at 20°C. Serial dilutions of standard antigen or sample in dilution buffer (10% FBS in PBS) were added to the plates, and the plates were incubated for 2 h at 20°C. After the plates were washed, biotin-conjugated anti-mouse IgE and streptavidin-conjugated horseradish peroxidase (SAv-HRP) were added to the plates, and the plates were incubated for 1 h at 20°C. Finally, the tetramethylbenzidine (TMB) substrate was added to the plates, and after 15 min of incubation in the dark, 2 N H_2_SO_4_ was added to stop the reaction. The optical density was measured at 450 nm on an automated ELISA reader.

### Rhodamine 123 efflux assay

MCF-7 and MCF-7/PAC cells were treated with SH003 (0–500 µg/ml) or nicardipine (positive control, 12.5 μM) for 24 h and incubated for another 1 h with 1 μg/ml of rhodamine 123. Accumulation of rhodamine 123 in the cells was analyzed by flow cytometry.

### Statistical analysis

All experiments were performed in triplicate. The data for the MTT assay, clonogenic survival assay, and ELISA assay are expressed as the mean ± standard deviation (SD). The standard deviations for all of the measured biological parameters are displayed in the appropriate figures. Student’s *t*-test was used for single variable comparisons, and a *P*-value of < 0.05 was considered statistically significant.

## Results

### SH003 suppresses the growth of MCF-7/PAC cells

**Figure 1:**
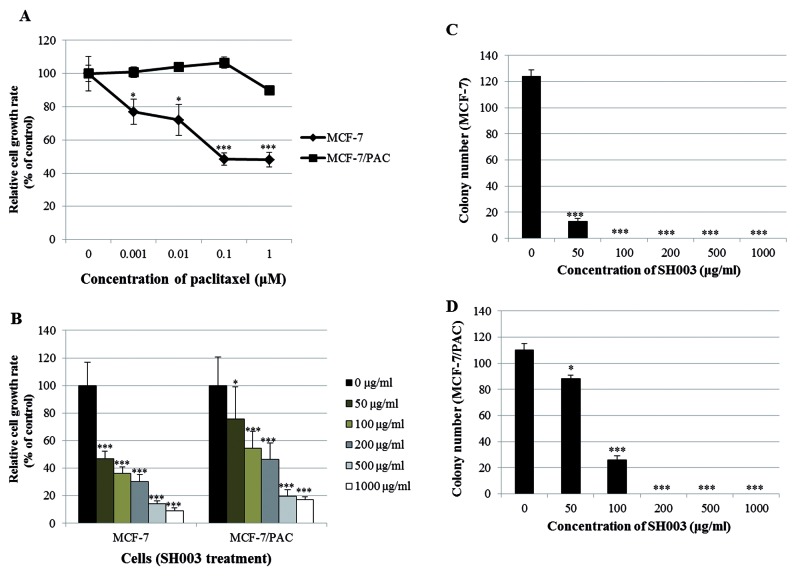
SH003 suppresses the growth of MCF-7/PAC cells (**A**) MCF-7 and MCF-7/PAC cells were treated with different doses of paclitaxel (0–1 μM). After 48 h, the relative cell growth rate was assessed by MTT assay. (**B**) MCF-7 and MCF-7/PAC cells were treated with different doses of SH003 (0–1000 μg/ml). After 72 h, the relative cell growth rate was assessed by MTT assay. The growth rate of the vehicle-treated cells was set to 100%, and the relative decrease in cell viability resulting from treatment with the compound was expressed as a percentage of the control. The data are shown as the means of three independent experiments (error bars denote SD); *P<0.05, ***P<0.001. (**C** and **D**) MCF-7 and MCF-7/PAC cells were seeded in six-well culture plates at a density of 5 × 10^2^ cells/well. After overnight incubation, the cells were treated with different concentrations of SH003 (0–1000 μg/ml) and maintained for 10 days at 37°C. Finally, the plates were stained with hematoxylin, and the colony numbers were counted. The data shown are representative of three independent experiments that gave similar results.

Before investigating the effect of SH003 on the growth of paclitaxel-resistant cells, we determined whether MCF-7/PAC cells display resistance to paclitaxel. Figure 1A demonstrates that paclitaxel decreased the growth rate of MCF-7 cells, while it did not affect the growth rate of MCF-7/PAC cells, confirming that the MCF-7/PAC cell line is paclitaxel resistant. Figure 1B indicates that SH003 significantly inhibited the proliferation of both MCF-7 and MCF-7/PAC cells in a dose-dependent manner after 72 h of treatment. This growth inhibition induced by SH003 was verified by microscopic observation (Supplementary Figure S1). SH003 also induced morphological changes in these cells (Supplementary Figure S1). These results indicate that SH003 suppresses the growth of paclitaxel-resistant cells.

### SH003 inhibits clonogenic survival of MCF-7/PAC cells

Next, we investigated the effect of SH003 on the clonogenic survival of MCF-7/PAC cells using a colony formation assay. As shown in Figure 1C and D, SH003 significantly inhibited colony formation in both MCF-7 and MCF-7/PAC cells in a dose-dependent manner. The effect of SH003 was stronger in MCF-7 cells than in MCF-7/PAC cells, as evidenced by the relative absence of colony formation in MCF-7 cells and the presence of colonies in MCF-7/PAC cells with 50 μg/ml treatment. These results indicate that MCF-7/PAC cells are drug resistant and that SH003 overcomes this resistance.

### SH003 induces apoptosis in MCF-7/PAC cells

**Figure 2:**
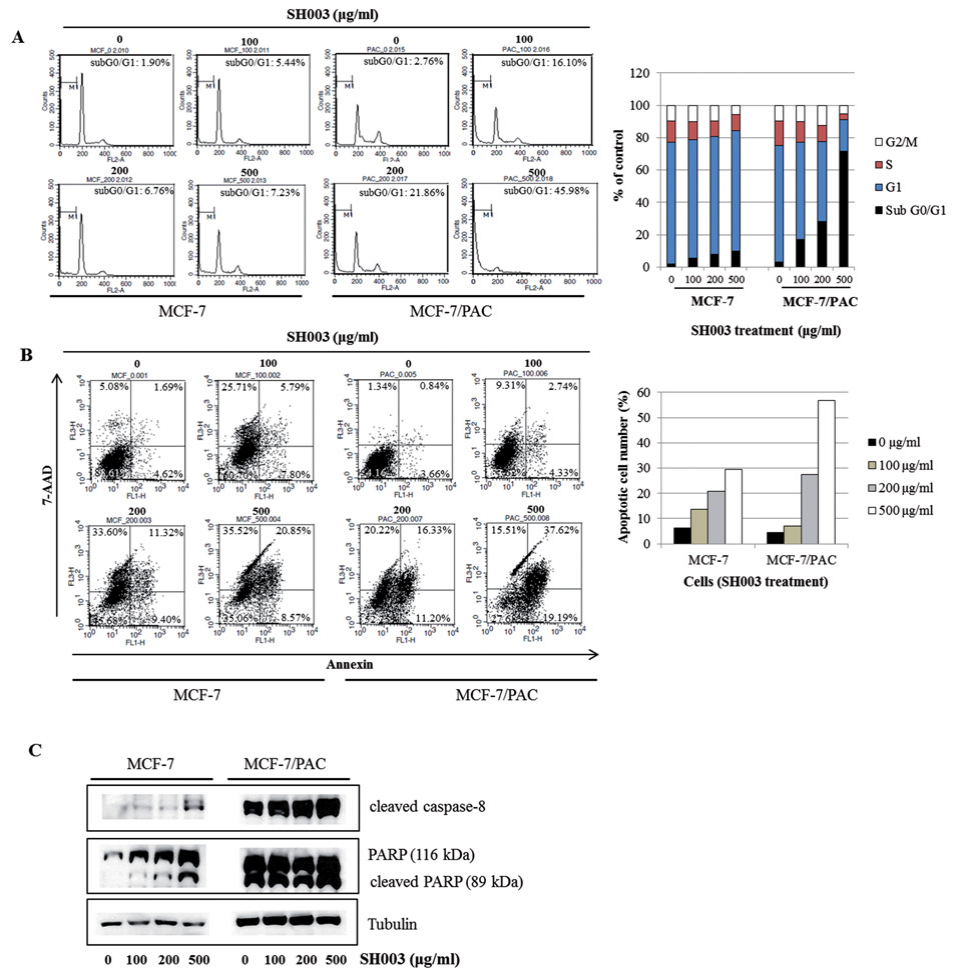
SH003 induces apoptosis in MCF-7/PAC cells (**A**) MCF-7 and MCF-7/PAC cells were treated with SH003 (0–500 μg/ml) and fixed 72 h later for flow cytometry. Propidium iodide-labeled nuclei were analyzed for DNA content. The sub-G_0_/G_1_ apoptotic population was quantified. (**B**) MCF-7 and MCF-7/PAC cells were treated with SH003 (0–500 μg/ml) and harvested after 72 h. Cells were stained with 7-AAD and annexin V-FITC. Apoptotic cell death was analyzed with a BD FACSCalibur flow cytometer using the FL1 and FL3 channels. (**C**) MCF-7 and MCF-7/PAC cells were treated with SH003 (0–500 μg/ml) for 24 h. Whole cell lysates were analyzed by Western blotting with anti-cleaved caspase-8, anti-PARP, and anti-tubulin antibodies. The data shown are representative of three independent experiments that gave similar results.

To investigate whether SH003 inhibits cell proliferation by promoting changes in cell cycle progression, the effect of SH003 on the cell cycle profile was assessed in MCF-7 and MCF-7/PAC cells. For this purpose, cells were treated with SH003 (0–500 µg/ml) for 72 h and then analyzed for cell cycle stage by flow cytometry. The results demonstrated that SH003 induced an increase in the sub-G_0_/G_1_ apoptotic population in both cells (Figure 2A). In the annexin V assays, we found that SH003 increased the number of apoptotic cells in both cell lines (Figure 2B). Consistent with these results, we also found that SH003 altered the apoptotic protein machineries; SH003 up-regulated the levels of cleaved caspase-8 and PARP in both cell lines (Figure 2C). Therefore, our data indicate that SH003 induces apoptosis in chemotherapy-resistant breast cancer cells.

### SH003 down-regulates MDR1 expression in MCF-7/PAC cells

**Figure 3:**
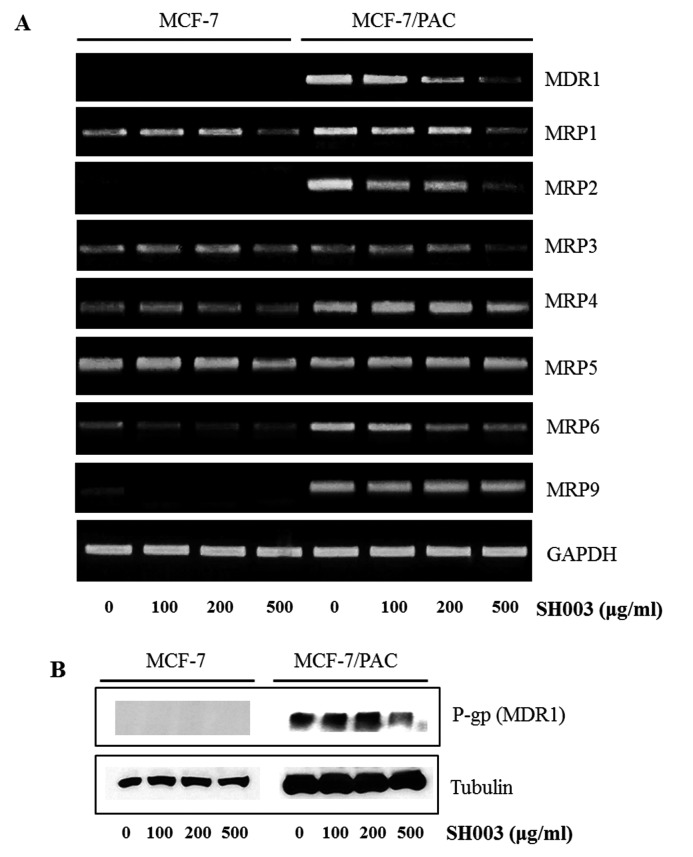
SH003 down-regulates MDR1 expression in MCF-7/PAC cells (**A**) MCF-7 and MCF-7/PAC cells were treated with SH003 (0–500 μg/ml) for 24 h, and the mRNA levels of MDR1 and MRPs were measured by RT-PCR. The data shown are representative of three independent experiments that gave similar results. (**B**) MCF-7 and MCF-7/PAC cells were treated with SH003 (0–500 μg/ml) for 24 h. Whole cell lysates were analyzed by Western blotting with anti-MDR1 and anti-tubulin antibodies. The data shown are representative of three independent experiments that gave similar results.

Because P-gp (MDR1) and MRPs are important contributors to drug resistance, we checked whether SH003 regulates their expression. RT-PCR demonstrated that SH003 reduced the mRNA expression of MDR1, MRP1-6, and MRP9 (Figure 3). Western blot analysis indicated that P-gp (MDR1) was not induced in MCF-7 cells in the presence or absence of SH003, while SH003 decreased the protein expression of P-gp (MDR1) in MCF-7/PAC cells (Figure 3). These results indicate that SH003 inhibits drug resistance by reducing the expression of drug resistance proteins in MCF-7/PAC cells.

### SH003 recovers accumulation of rhodamine 123 in MCF-7/PAC cells

**Figure 4:**
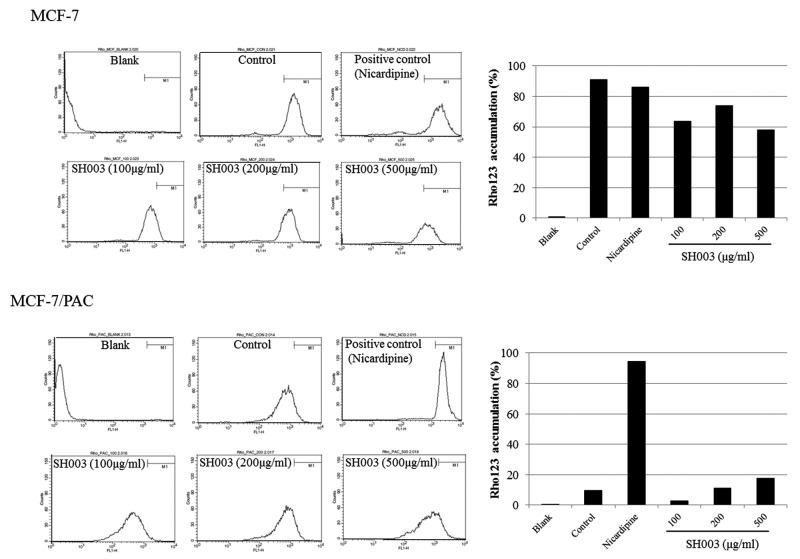
SH003 recovers the accumulation of rhodamine 123 in MCF-7/PAC cells MCF-7 and MCF-7/PAC cells were treated with SH003 (0–500 μg/ml) or nicardipine (positive control, 12.5 μM) for 24 h and incubated for another 1 h with rhodamine 123. Rhodamine 123 accumulation was analyzed by flow cytometry.

Because drug resistance is associated with drug efflux by the cells, we performed a rhodamine 123 assay to investigate whether SH003 recovers drug accumulation in MCF-7/PAC cells. As expected, rhodamine 123 accumulation was high in the MCF-7 cell line, which is a drug-sensitive cell line (Figure 4). In contrast, rhodamine 123 was ejected from the drug-resistant MCF-7/PAC cells, but SH003 recovered rhodamine 123 accumulation (Figure 4). These results indicate that SH003 inhibits drug resistance by suppressing drug efflux in MCF-7/PAC cells.

### SH003 inhibits the STAT3 signaling pathway, resulting in suppression of drug resistance

**Figure 5:**
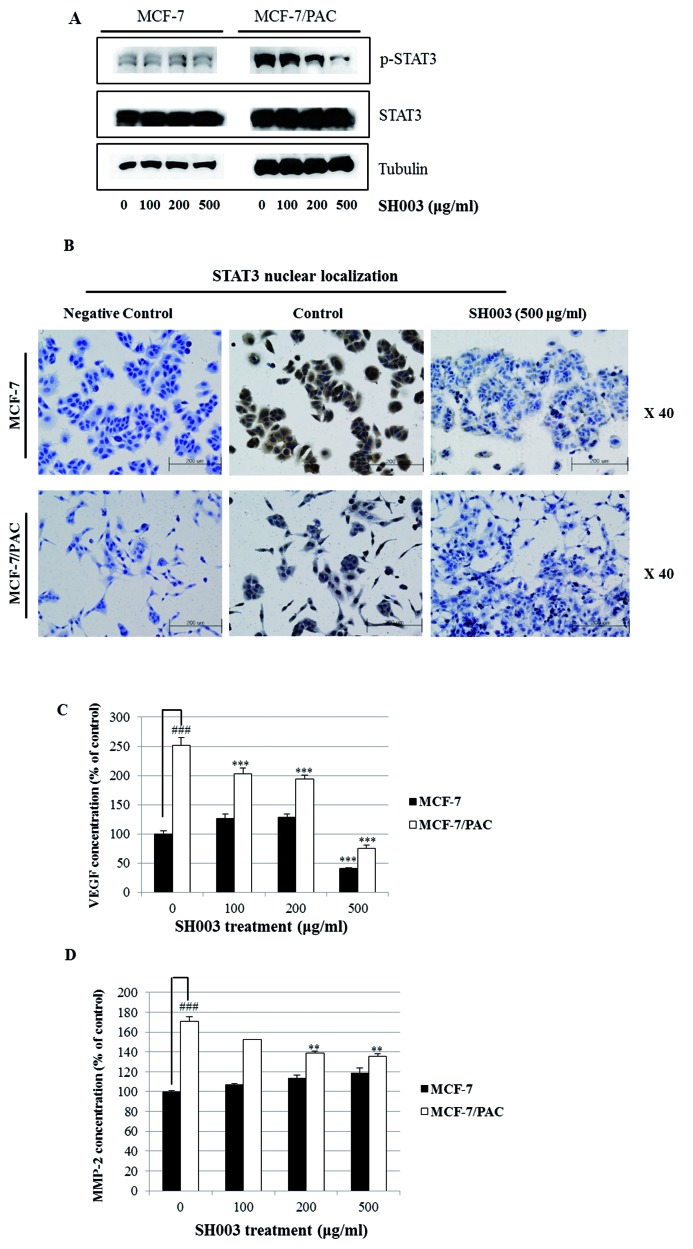
SH003 inhibits the STAT3 signaling pathway (**A**) MCF-7 and MCF-7/PAC cells were treated with SH003 (0–500 μg/ml) for 24 h. Whole cell lysates were analyzed by Western blotting with anti-p-STAT3, anti-STAT3, and anti-tubulin antibodies. The data shown are representative of three independent experiments that gave similar results. (**B**) MCF-7 and MCF-7/PAC cells were treated with SH003 (500 μg/ml) for 24 h and submitted to immunocytochemistry for detection of nuclear STAT3. The data shown are representative of three independent experiments that gave similar results. (**C** and **D**) MCF-7 and MCF-7/PAC cells were treated with SH003 (0–500 μg/ml) for 24 h, and the intracellular VEGF and MMP-2 concentration was measured by ELISA. The data are shown as the means of three independent experiments (error bars denote SD); **P<0.01, ***P<0.001.

**Figure 6:**
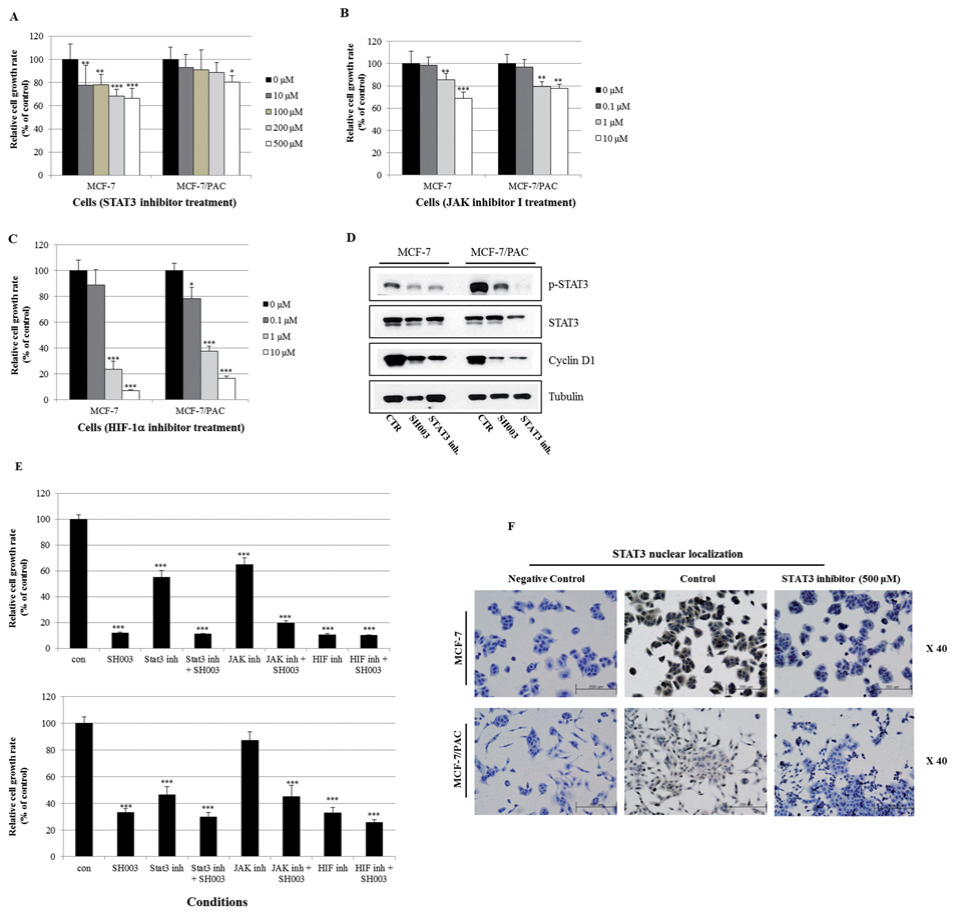
Inhibition of STAT3 signaling induces the growth suppression of MCF-7/PAC cells (**A**–**C**) MCF-7 and MCF-7/PAC cells were treated with an STAT3 inhibitor (S3I-201, 0–500 µM), JAK inhibitor I (0–10 µM), and an HIF-1α inhibitor (EF-24, 0–10 µM) for 72 h. The relative cell growth rate was measured by MTT assay. The growth rate of the vehicle-treated cells was set to 100%, and the relative decrease in cell viability resulting from treatment with the compound was expressed as a percentage of the control. The data are shown as the means of three independent experiments (error bars denote SD); *P<0.05, **P<0.01, ***P<0.001. (**D**) MCF-7 and MCF-7/PAC cells were treated with SH003 (500 μg/ml) and an STAT3 inhibitor (S3I-201, 500 µM) for 24 h. Whole cell lysates were analyzed by Western blotting with anti-p-STAT3, anti-STAT3, anti-cyclin D1, and anti-tubulin antibodies. (**E**) MCF-7 and MCF-7/PAC cells were treated with STAT3 inhibitor (500 µM), JAK inhibitor I (10 µM), and an HIF-1α inhibitor (10 µM) for 72 h in the absence or presence of SH003 (500 μg/ml). The relative cell growth rate was measured by MTT assay. The growth rate of the vehicle-treated cells was set to 100%, and the relative decrease in cell viability resulting from treatment with the compound was expressed as a percentage of the control. The data are shown as the means of three independent experiments (error bars denote SD); *P<0.05, **P<0.01, ***P<0.001. (**F**) MCF-7 and MCF-7/PAC cells were treated with STAT3 inhibitor (S3I-201, 500 µM) for 24 h and submitted to immunocytochemistry for detection of nuclear STAT3. The data shown are representative of three independent experiments that gave similar results.

Since STAT3 is related to oncogenic signaling, and it is known that STAT3 deactivation reverses chemotherapeutic resistance [20], we investigated whether SH003 inhibits the STAT3 signaling pathway in MCF-7/PAC cells. We found that SH003 reduced the expression of p-STAT3 in MCF-7/PAC cells (Figure 5A). Immunocytochemical staining indicated that SH003 decreased the nuclear localization of STAT3 in both MCF-7 and MCF-7/PAC cells (Figure 5B). An ELISA demonstrated that SH003 decreased the production of intracellular VEGF and MMP-2, which are STAT3 target genes in MCF-7/PAC cells (Figure 5C and D). Moreover, an STAT3 inhibitor (S3I-201), JAK inhibitor I, and HIF-1α inhibitor (EF-24) induced cell growth inhibition in both MCF-7 and MCF-7/PAC cells, as shown in Figure 6A–C. Figure 6D demonstrates that both SH003 (500 µg/ml) and an STAT3 inhibitor (S3I-201, 500 µM) reduced the expression of p-STAT3 and cyclin D1 (an STAT3 target gene) in MCF-7/PAC cells. Figure 6E indicated that SH003 induced strong inhibitory effect on cell growth when cotreated with STAT3 inhibitor, JAK inhibitor I, and HIF-1α inhibitor. Figure 6F demonstrated that STAT3 inhibitor decreased the nuclear localization of STAT3 in both MCF-7 and MCF-7/PAC cells. These results indicate that SH003 suppresses drug resistance by inhibiting the STAT3 signaling pathway in MCF-7/PAC cells. Therefore, STAT3 may be a promising therapeutic target for overcoming drug resistance in breast cancer.

## Discussion

The development of MDR to chemotherapy remains a major challenge in the treatment of cancer [21]. Thus, overcoming MDR is an important endeavor that is necessary to increase the overall survival of cancer patients [22]. Natural herbal medicine, such as SH003, is the object of intense research for their potential to reverse MDR, which would result in successful chemotherapy treatments. In the present study, the mechanism by which SH003 overcomes drug resistance was investigated in breast cancer cells. The aim of the study was to determine whether SH003 might be a useful compound for reversing drug resistance. For this purpose, we used paclitaxel-resistant cells, MCF-7/PAC.

SH003 significantly inhibited the growth of both MCF-7 and MCF-7/PAC cells. The clonogenic survival assay revealed that SH003 decreased anchorage-dependent colony formation of both cell lines in a dose-dependent manner. This growth inhibition was associated with an increase in the sub-G_0_/G_1_ apoptotic population in MCF-7 and MCF-7/PAC cells. SH003 increased the number of apoptotic cells in a dose-dependent manner, as assessed by annexin V assays. Moreover, SH003 induced apoptosis via a caspase-dependent apoptosis pathway, as shown by the cleavage of caspase-8 and PARP. We did not detect caspase-3, which is important in the apoptosis pathway, because MCF-7 cells do not contain caspase-3 due to a genomic deletion [23]. Our results indicate that SH003 contains strong apoptotic properties. Activation of apoptotic caspases induces inactivation or activation of substrates, leading to a signaling cascade, and the initiation of this signaling pathway permits the controlled destruction of cellular components [24]. SH003-induced apoptosis seems to be mediated by the activation of caspase-8.

SH003 reduced the mRNA expression of MDR1 and MRPs, as well as the protein expression of P-gp (MDR1). SH003 also inhibited drug efflux from the MCF-7/PAC paclitaxel-resistant cells in a dose-dependent manner, as revealed by the rhodamine 123 efflux assay. These results indicate that SH003 overcomes drug resistance. P-gp is a transmembrane glycoprotein that reduces intracellular drug concentrations by pumping drugs out of the cells [20]. Regulation of P-gp expression is important to avoid drug resistance; overexpression of P-gp induces resistance to chemotherapeutic reagents. SH003 appears to inhibit P-gp-mediated drug resistance in MCF-7/PAC cells.

Interestingly, SH003 reduced the expression of p-STAT3, suggesting that it negatively regulates the STAT3 pathway in MCF-7/PAC cells. SH003 inhibited nuclear localization of STAT3 in MCF-7/PAC cells, as revealed by immunocytochemistry. SH003 inhibited the production of VEGF and MMP-2, which are STAT3 target genes, in MCF-7/PAC cells, as revealed by the ELISA assay. The STAT3 inhibitor (S3I-201), JAK inhibitor I, and the HIF-1α inhibitor (EF-24) decreased the growth of MCF-7/PAC cells. These results clearly indicate that SH003 induces growth-suppressive activity and overcomes drug resistance by inhibiting the STAT3 signaling pathway. STAT3 is a transcription factor that regulates gene expression in response to various cellular stimuli and plays an important role in cell growth and apoptosis. STAT3 usually acts as a tumor promoter, although its role as a tumor suppressor has been recently reported [25,26]. STAT3 accelerates cell proliferation and angiogenesis, inhibits apoptosis, and drives invasion and metastasis [27–30]. STAT3 in melanoma tumors is associated with poor prognosis [27–30]. Constitutive STAT3 phosphorylation is mediated by several upstream kinases (Jak and Src) and is thought to be a key component of the oncogenic process [31,32]. The phytoestrogen resveratrol is known to inhibit STAT3 signaling and induce apoptosis in malignant cells containing activated STAT3 [33]. The VEGF promoter contains various transcription factor binding sites, including sites for STAT3 [28, 29] and HIF-1 [34]. The physical interaction of STAT3 with HIF-1 controls VEGF transcriptional activation by binding to the VEGF promoter [35]. Constitutive activation of STAT3 has been shown to confer resistance to chemotherapy-induced apoptosis in some malignancies [20]. Additionally, some researchers have demonstrated that multidrug resistance was consistent with *STAT3* mRNA overexpression in cisplatin-resistant lung cancer cells [20]. The activity of STAT3 is usually higher in MDR malignancies, and inhibition of STAT3 activity might reverse chemoresistance [20]. Constitutive activation of the STAT3 pathway could be an early indicator of drug resistance. Therefore, SH003 seems to reverse drug resistance by inhibiting STAT3 signaling, which indicates that SH003 could be a useful natural therapy that overcomes drug resistance. SH003 could be a promising therapy for the treatment and prevention of breast cancer.

## Supporting information
