# Supplementary figures and images for: SH003 reverses drug resistance by blocking signal transducer and activator of transcription 3 (STAT3) signaling in breast cancer cells
Source: Biosci Rep. 2017 Nov 15;37(6):BSR20170125. doi: 10.1042/BSR20170125 (PMC5686394; doi:10.1042/BSR20170125)

## MCF-7

---

**EtOH 30%**

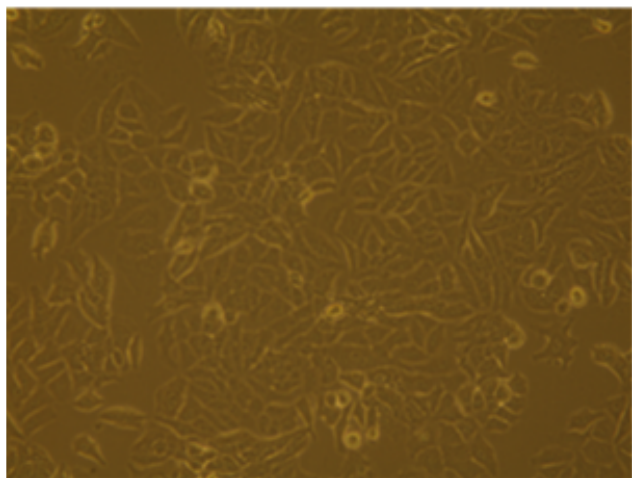

**SH003 (100 µg/ml)**

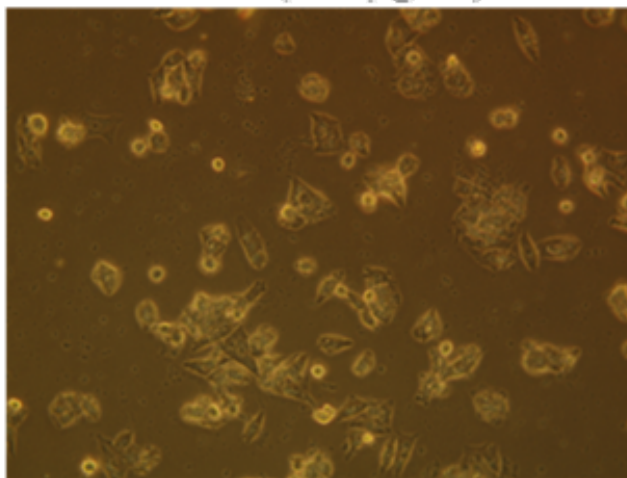

**SH003 (200 µg/ml)**

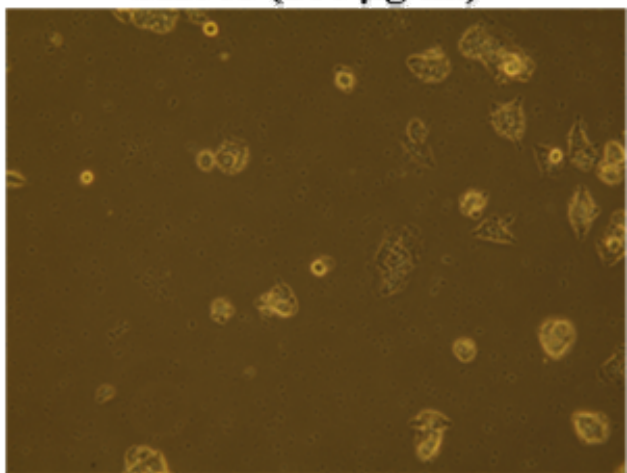

**SH003 (500 µg/ml)**

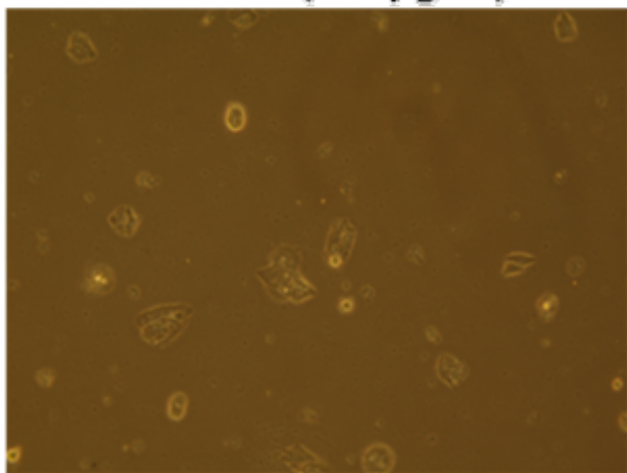

**EtOH 30%**

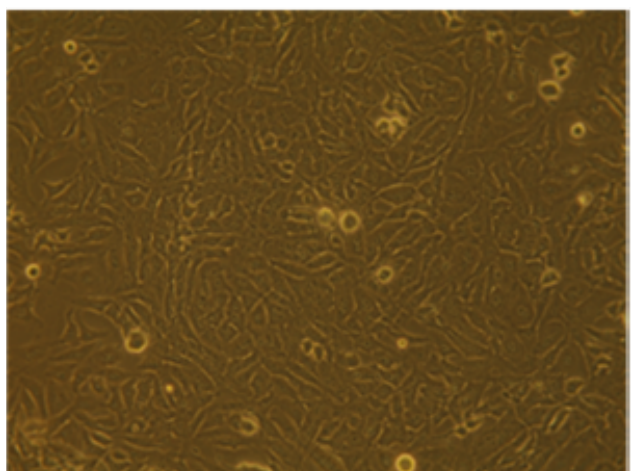

**SH003 (100 µg/ml)**

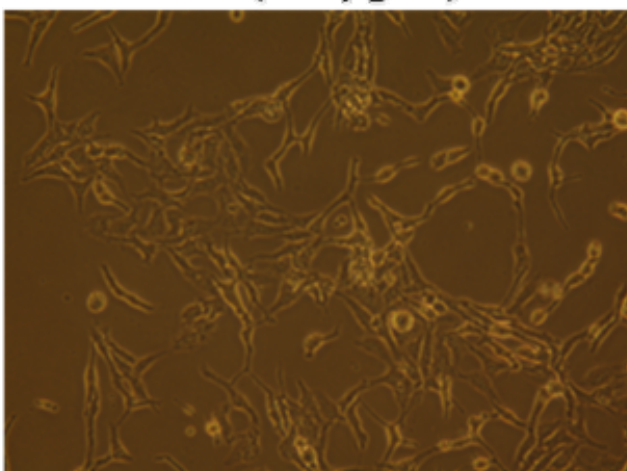

**SH003 (200 µg/ml)**

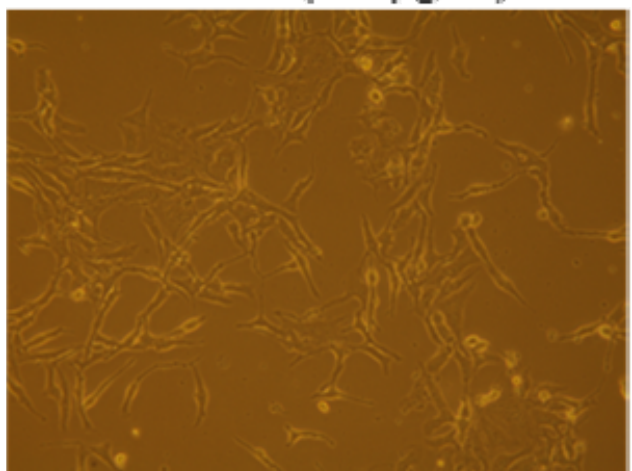

**SH003 (500 µg/ml)**

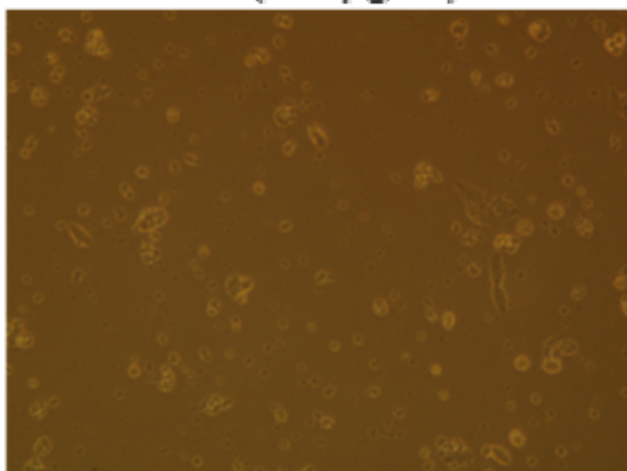

## MCF-7/PAC

---

Supplement: Supplementary file 1 [file bsr20170125_Supp1.pdf]
